# The Remarkable Metabolism of *Vickermania ingenoplastis*: Genomic Predictions

**DOI:** 10.3390/pathogens10010068

**Published:** 2021-01-14

**Authors:** Fred R. Opperdoes, Anzhelika Butenko, Alexandra Zakharova, Evgeny S. Gerasimov, Sara L. Zimmer, Julius Lukeš, Vyacheslav Yurchenko

**Affiliations:** 1 De Duve Institute, Université Catholique de Louvain, 1200 Brussels, Belgium; 2 Life Science Research Centre, Faculty of Science, University of Ostrava, 710 00 Ostrava, Czech Republic; anzhelika.butenko@paru.cas.cz (A.B.); alexandraz.6946@gmail.com (A.Z.); 3 Biology Centre, Institute of Parasitology, Czech Academy of Sciences, 370 05 České Budějovice (Budweis), Czech Republic; jula@paru.cas.cz; 4 Faculty of Biology, M.V. Lomonosov Moscow State University, 119991 Moscow, Russia; jalgard@gmail.com; 5 Martsinovsky Institute of Medical Parasitology, Tropical and Vector Borne Diseases, Sechenov University, 119435 Moscow, Russia; 6 Department of Biomedical Sciences, University of Minnesota Medical School, Duluth Campus, Duluth, MN 558812, USA; szimmer3@d.umn.edu; 7 Faculty of Science, University of South Bohemia, 370 05 České Budějovice (Budweis), Czech Republic

**Keywords:** *Vickermania ingenoplastis*, *Phytomonas*, metabolism, genome sequencing

## Abstract

A recently redescribed two-flagellar trypanosomatid *Vickermania ingenoplastis* is insensitive to the classical inhibitors of respiration and thrives under anaerobic conditions. Using genomic and transcriptomic data, we analyzed its genes of the core metabolism and documented that subunits of the mitochondrial respiratory complexes III and IV are ablated, while those of complexes I, II, and V are all present, along with an alternative oxidase. This explains the previously reported conversion of glucose to acetate and succinate by aerobic fermentation. Glycolytic pyruvate is metabolized to acetate and ethanol by pyruvate dismutation, whereby a unique type of alcohol dehydrogenase (shared only with *Phytomonas* spp.) processes an excess of reducing equivalents formed under anaerobic conditions, leading to the formation of ethanol. Succinate (formed to maintain the glycosomal redox balance) is converted to propionate by a cyclic process involving three enzymes of the mitochondrial methyl-malonyl-CoA pathway, via a cyclic process, which results in the formation of additional ATP. The unusual structure of the *V. ingenoplastis* genome and its similarity with that of *Phytomonas* spp. imply their relatedness or convergent evolution. Nevertheless, a critical difference between these two trypanosomatids is that the former has significantly increased its genome size by gene duplications, while the latter streamlined its genome.

## 1. Introduction

Trypanosomatids (Euglenozoa: Kinetoplastea: Trypanosomatidae) are parasites of annelids, arthropods, plants, and vertebrates, with leeches and insects serving as transmission vectors. The best-known trypanosomatids are dixenous (=circulating between two hosts, *Leishmania*, *Phytomonas*, and *Trypanosoma* spp.) and infect vertebrates, including humans and plants [1,2]. Nevertheless, the vast majority of species are monoxenous (=confined to a single host, usually an insect) [3]. Monoxenous Trypanosomatidae are ancestral and significantly more diverse [4,5,6].

Virtually all trypanosomatids are mono-flagellated (including an “amastigote” stage in some life cycles, which has an extremely short flagellum [7]) and use their flagella for attachment, movement, production of the extracellular vesicles, and environment sensing [8,9,10,11]. The only exception to this rule is members of the recently established genus *Vickermania*, *V. ingenoplastis* and *V. spadyakhi* [12]. These flagellates are adapted to the life in the fly midgut, to which they do not attach. Instead, they move constantly, resisting the midgut peristaltic flow within the fly host. To this end, *Vickermania* has disconnected duplication of flagella from the cell cycle and developed a mechanism to join the newly growing flagellum with the old one. As such, these trypanosomatids possess two flagella for a significant period of their life cycle.

In contrast to most other trypanosomatids, the metabolic activity of the *V. ingenoplastis* mitochondrion is strongly reduced. Cytochrome-mediated respiration was found to be missing, and energy metabolism to be based mainly on the fermentative glycolysis with acetate and ethanol as the major end-products and propionate and succinate as minor products. Interestingly, the switch from aerobic to anaerobic conditions had minimal effects [13]. Several enzymes of the Krebs cycle were not detected, and the presence of fumarate reductase activity was interpreted to indicate that CO_2_ fixation and reverse flux through part of the Krebs cycle enables growth under anaerobic conditions [14]. This would be a unique feature for trypanosomatid parasites, which are generally considered to be (strictly) aerobic [15]. Respiration was not inhibited by cyanide, while malonate and salicyl-hydroxamic acid strongly reduced succinate oxidation. Taken together, these observations demonstrated that *V. ingenoplastis* lacks the mitochondrial respiratory complex IV, has an inactive complex III, while complex II and an alternative oxidase seem to be functional [16]. All these biochemical observations were experimental. Armed with the recently obtained genomic data for this species [17], we reanalyzed the metabolic potential of *V. ingenoplastis*, explain some earlier contradictory observation and provide a coherent framework for its unique metabolism. In particular, we documented that *V. ingenoplastis* has lost genes coding for the subunits of the respiratory complexes III and IV, increased a number of genes involved in carbohydrate metabolism, and lost the capacity to oxidize fatty acids and a number of aromatic and branched amino acids.

## 2. Results

### 2.1. Global Comparison between *Vickermania ingenoplastis* and *Leishmania major Genomes*

The genome of *V. ingenoplastis* was reassembled *de novo* using different programs, resulting in its significant improvement from the previously published version [17]. The obtained assembly is 34.3 Mbp (in contrast to the original 35.3 Mbp, hereafter the data of the previous assembly are given in parentheses) in 241 scaffolds (340) with N_50_ of 591 kb (376 kb) and the longest contig of 2.4 Mb (1.6 Mb). The new assembly is not only more contiguous but also more complete as judged by the higher total number of annotated protein-coding genes (9562 vs. 8619 reported previously) and improved benchmarking universal single-copy orthologs (BUSCO) scores. The percentage of complete BUSCOs increased from 84.6 to 93.1%, and only 4% of BUSCOs are missing compared to 11% in the previous assembly.

The genome of *V. ingenoplastis* contains high copy numbers of the glycolytic pathway genes, such as those encoding hexokinase, as well as glycosomal and cytosolic glyceraldehyde-3-phosphate dehydrogenases. This suggests that the irreversible loss of the mitochondrial oxidative phosphorylation in *V. ingenoplastis* (see below) may have led to severe metabolic stress, by which the expression of a number of the glycolytic enzymes was upregulated by massive amplification of the corresponding genes. This has led, on the one hand, to a reduction in unique metabolic genes (97 *Leishmania major* orthologues were not found, Table S1), and on the other hand, a compensation for this loss by an expansion of the genome from 6500 to 8500 genes typically present in Leishmaniinae to 9562 genes in *V. ingenoplastis*. Most of these genes resulted from gene duplications to form multi-copy gene families (for example, cell surface proteins amastins), the largest of them with copy numbers reaching 200 (Tables S2 and S3). Out of 486 proteins predicted to be involved in the general metabolism of trypanosomatids, 87 have no orthologues in the genome of *V. ingenoplastis*, while it encodes many metabolic genes in high copy numbers. The highest copy numbers were scored for glycosomal glyceraldehyde dehydrogenase (39), cytosolic glyceraldehyde dehydrogenase (38), and hexokinase (19). The genome of *V. ingenoplastis* contains an additional 3612 genes that are not present in the *L. major* genome. Excluding all the genes annotated as coding for hypothetical or viral origin proteins, 620 unique genes were retained (Table S4). Of note, the expression of metabolic genes was further confirmed by whole-transcriptome analysis (Table S2).

### 2.2. Mitochondrial Enzymes of Oxidative Phosphorylation: Complexes I and II

Genes encoding subunits of complexes I (NADH dehydrogenase) and II (succinate dehydrogenase) are invariably present (Table S5), suggesting that these complexes are fully operational. NADH dehydrogenase is a large complex, the subunits of which are encoded by both the mitochondrial and nuclear genomes [18,19]. *Vickermania ingenoplastis* appears to be endowed with most, if not all, of its essential nuclear-encoded subunits. In the case of succinate dehydrogenase, entirely encoded in the nuclear genome, its two catalytic subunits (the flavoprotein and FeS-containing subunits) are present, along with a large battery of auxiliary proteins identified in *Trypanosoma cruzi* [20].

### 2.3. Mitochondrial Enzymes of Oxidative Phosphorylation: Complexes III and IV

We identified numerous deletions in the maxicircle kDNA, affecting complexes III and IV. Interestingly, these deletions are accompanied by the corresponding ablation of the complementing nuclear-encoded subunits of these complexes (Table S5). For complex III, not only the mitochondrial-encoded cytochrome *b*, but also the ubiquinol cytochrome *c* reductase, cytochrome *c*_1_, the Rieske FeS protein, and complex III core protein [21] are absent. Of note, two members of the mitochondrial processing peptidase family that have been shown to be core subunits of the complex III (orthologues of the α-MMP or β-MMP) have been retained in *V. ingenoplastis*, likely reflecting their additional functions [22]. For complex IV (cytochrome oxidase), not only all three mitochondrial-encoded subunits (COXI, COXII, and COXIII) but also the nuclear-encoded subunits 4, 5, 6, 7 and 10 are absent, along with the cytochrome oxidase assembly protein COX15, involved in the synthesis of heme *a* [23] and the electron transport protein SCO1/SCO2, a metallochaperone, essential for the assembly of the catalytic core of cytochrome *c* oxidase [24]. Finally, the gene for cytochrome *c*, which transports electrons between complexes III and IV, is absent in the nuclear genome, whereas a gene for the alternative oxidase is prominently present. Interestingly, the same genes have been lost from the genome of *Phytomonas* spp. [25], further supporting the evolutionary relatedness of these genera [12].

We conclude that electrons entering the respiratory chain via NADH or succinate pass through the functional complexes I and II and reach coenzyme Q (ubiquinone), from where they are transferred to molecular oxygen via the alternative oxidase.

### 2.4. Mitochondrial Enzymes of Oxidative Phosphorylation: Complex V

Although complexes III and IV of the respiratory chain are completely missing, the ATP synthase (complex V) appears to be fully operational. The kinetoplast-encoded subunit of the F_1_F_0_ ATPase (subunit 6) is present in the maxicircle kDNA, along with all other nuclear-encoded subunits, including 11 auxiliary proteins [26] (Table S5).

### 2.5. Cytochrome o

The respiration of *V. ingenoplastis* was shown to be cyanide insensitive, with cytochrome (cyt) *o* being suggested as an alternative cyanide-resistant terminal oxidase [16]. However, this was not confirmed experimentally, and no gene for this enzyme has been described in trypanosomatids so far. The spectral characteristics of Cyt *o* inversely correlate with that of reduced Cyt *b.* This allows us to propose that the suspected Cyt *o* was a variant of Cyt *b*, not properly integrated into complex III, and subjected to auto-oxidization. As a consequence, this gene was lost over time. The alternative oxidase, encoded by six copies in *V. ingenoplastis*, might be responsible for the inhibition of respiration by SHAM [27]. Thus, far, an alternative oxidase has been documented in various euglenozoan lineages, including *Bodo*, *Trypanosoma*, *Phytomonas*, and *Angomonas,* as a single-copy gene [25,28,29].

### 2.6. Krebs Cycle

**Figure 1:**
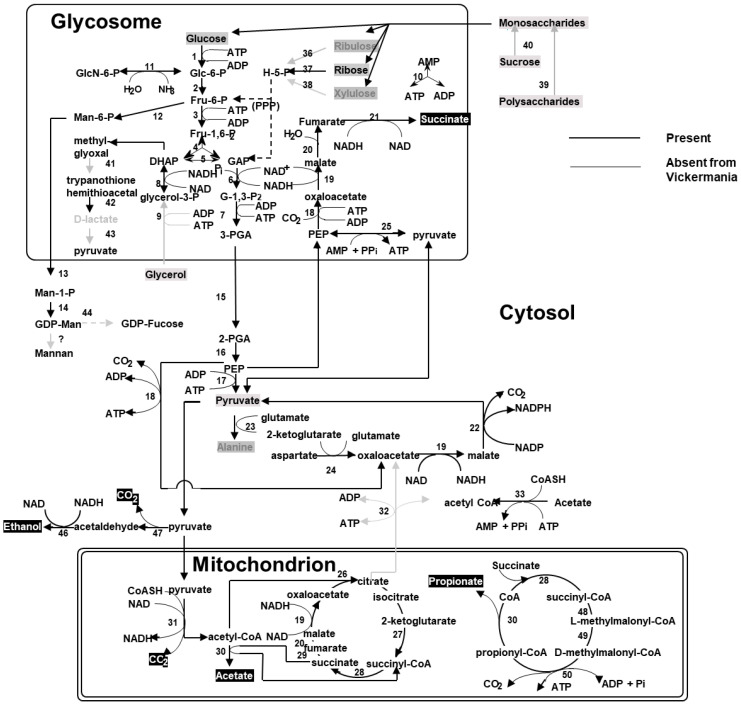
The pathways of core metabolism in *Vickermania ingenoplastis* as compared to that of *Leishmania major*. Boxed metabolites are nutrients (in gray) or end-products (in black). PPP, pentose-phosphate pathway. Enzymes: 1, hexokinase; 2, phosphoglucose isomerase; 3, phosphofructokinase; 4, fructosebisphosphate aldolase; 5, triosephosphate isomerase; 6, glyceraldehyde-3-phosphate dehydrogenase; 7, glycosomal phosphoglycerate kinase; 8, glycerol-3-phosphate dehydrogenase; 9 glycerol kinase; 10, glycosomal adenylate kinase; 11, glucosamine-6-phosphate deaminase; 12, mannose-6-phosphate isomerase; 13, phosphomannomutase; 14, GDP-mannose pyrophosphorylase; 15, phosphoglycerate mutase; 16, enolase; 17, pyruvate kinase; 18, phosphoenolpyruvate carboxykinase; 19, malate dehydrogenase; 20, fumarate hydratase; 21, NADH-dependent fumarate reductase; 22, malic enzyme; 23, alanine aminotransferase; 24, aspartate aminotransferase; 25, pyruvate phosphate di-kinase; 26, citrate synthase; 27, 2-ketoglutarate dehydrogenase; 28, succinyl-CoA ligase; 29, succinate dehydrogenase; 30, acetate:succinate CoA transferase; 31, pyruvate dehydrogenase; 32, citrate lyase; 33, acetyl-CoA synthetase; 36, ribulokinase; 37, ribokinase, 38, xylulokinase; 39, glucoamylase; 40, invertase; 41, glyoxalase I; 42, glyoxalase II; 43, D-lactate dehydrogenase; 44, GDP-mannose 4,6-dehydratase and GDP-L-fucose synthase; 45, phosphoacetylglucosamine mutase and glucosamine-1-phosphate acetyl transferase/UDP-N-acetylglucosamine transferase; 46, alcohol dehydrogenase GroES-like domain/zinc-binding dehydrogenase; 47, pyruvate decarboxylase; 48, methylmalonyl-CoA epimarase; 49, methylmalonyl-CoA mutase; 50, propionyl-CoA carboxylase. Modified from [25].

Our genome analysis indicates that, except for the catabolic NAD-dependent isocitrate dehydrogenase, which has been replaced by an anabolic NADP-dependent isoenzyme, all other Krebs cycle enzymes (citrate synthase, aconitase, isocitrate dehydrogenase, 2-oxoglutarate dehydrogenase, succinyl-CoA ligase, succinate dehydrogenase, fumarate hydratase and malate dehydrogenase) and subunits of the pyruvate dehydrogenase complex are present. The presence of an NADP-dependent isocitrate dehydrogenase, rather than an NAD-dependent enzyme, resembles the situation in other trypanosomatids and predicts that the Krebs cycle in *V. ingenoplastis* functions not as a real cycle [30,31], but CO_2_ fixation and reverse flux through a part of the cycle may endow this flagellate with aerobic fermentation (Figure 1).

### 2.7. Carbohydrate Metabolism

The *Vickermania ingenoplastis* genome contains all genes of the glycolytic pathway, with many of them predicted to possess a peroxisome targeting signal (Table S6). In addition, a whole battery of peroxisome assembly factors is present. Both observations strongly imply the presence of glycosomes, as in all other trypanosomatids [32]. The high copy numbers for several of the glycolytic genes suggest that the corresponding enzymes are abundantly present. This agrees with previous observations of numerous microbodies (likely, glycosomes) in the electron micrographs of *V. ingenoplastis* [14] and the fact that glucose consumption via the glycolytic pathway is *Vickermania*’s principal source of energy [13]. Pyruvate, the end-product of this pathway, is oxidized in the mitochondrion by the pyruvate dehydrogenase complex and the resulting acetyl-CoA is excreted in the form of acetate, the major end-product of *V. ingenoplastis* [13]. We have also documented the presence of Zn-containing alcohol dehydrogenase in the analyzed genome (Figure 1), an enzyme previously reported only from *Phytomonas* sp. [33]. This enzyme is likely responsible for the production of ethanol, since classical alcohol dehydrogenases are absent from all trypanosomatids.

Propionate, the other end-product of carbohydrate metabolism, is normally not produced by trypanosomatids, but only by some anaerobic eukaryotes [34] and *V. ingenoplastis* [13]. Moreover, a ratio of excreted end-products (succinate and propionic acid over acetate, two to one) is reminiscent of that observed in many propionic-acid producing anaerobic eukaryotes, such as the liver fluke *Fasciola hepatica*. A ratio of 2:1 is required to maintain the intracellular redox balance. This kind of fermentation is called “malate dismutation” [35]. Cytosolic malate (or pyruvate) is partly oxidized (*via* pyruvate) to acetate and partly reduced to succinate and propionate in the mitochondrion [34,36]. Succinate production requires the presence of the enzyme fumarate reductase, which, in most anaerobic eukaryotes, is a membrane-bound mitochondrial enzyme, similar or identical to the mitochondrial succinate dehydrogenase, except that it uses rhodoquinone, rather than ubiquinone, as the redox carrier, by which the enzyme is able to run in the reverse direction. In trypanosomatids, the presence of rhodoquinone has never been reported. Thus, the functioning of such a rodoquinone-dependent fumarate reductase was considered unlikely [36]. However, trypanosomatids are unique in that succinate can be readily formed within the highly reduced matrix of the glycosomes [32]. It is formed from phosphoenolpyruvate and CO_2_ via oxaloacetate, malate, and fumarate by the glycosomal NADH-dependent fumarate reductase (Figure 1). The two moles of NAD^+^ formed in this pathway serve to equilibrate the glycosomal NAD^+^/NADH balance. Anaerobically growing *V. ingenoplastis* now has the option to either excrete the succinate, as do all other trypanosomatids or to transport succinate into the mitochondrion, where it is converted to propionate in a cyclic process that contains three enzymes of the mitochondrial methyl-malonyl-CoA pathway (the “propionate cycle”). This way, the formation of each mole of propionate is accompanied by the formation of 1 mole of ATP by substrate-level phosphorylation.

Thus, under anaerobic conditions, the glucose oxidation in *Vickermania ingenoplastis* leads to the formation of 1 mole of pyruvate in the cytosol and 1 mole of succinate in the glycosome, along with 2 moles of ATP by substrate-level phosphorylation. Half of the pyruvate is transported into the mitochondrion and oxidized by the pyruvate dehydrogenase complex to acetyl-CoA and (*via* the acetate: succinate CoA transferase—succinyl-CoA ligase cycle) acetate with the net synthesis of an additional 1/2 mole of ATP. The other half of the pyruvate is decarboxylated by a cytosolic pyruvate decarboxylase to acetaldehyde and then reduced to 1/2 mole of ethanol. The latter reaction serves to reoxidize the NADH produced in the mitochondrial pyruvate dehydrogenase reaction. Thus, here, redox balance is maintained by a mechanism that should be called “pyruvate dismutation”. Glycosomal succinate is either excreted or decarboxylated to propionate in the mitochondrion with the concomitant formation of 1 mole of ATP. According to this scheme, the overall oxidation of 1 mole of glucose leads to the formation of 0.5 mole ethanol, 0.5 mole acetate, 1 mole of (succinate + propionate), and 3.5 moles of ATP. The anaerobic production of 3.5 moles of ATP per 1 mole of glucose consumed must be the explanation of why *V. ingenoplastis* is able to survive and grow under completely anaerobic conditions. In this respect, it is important to note that the bloodstream form of the African trypanosomes produce only 2 moles ATP per 1 mole of consumed glucose aerobically, while under anaerobic conditions (or when oxygen consumption is inhibited by SHAM) when only 1 mole ATP per 1 mole glucose is produced, the trypanosomes die [37]. No L-lactate is formed because a gene for L-lactate dehydrogenase is missing.

### 2.8. Hexose-Monophosphate Shunt and Gluconeogenesis

The enzymes of the hexose monophosphate pathway, as well as those involved in gluconeogenesis (except for the fructose-1,6-bisphosphatase), are present in the *V. ingenoplastis* genome (Table S2). There is no evidence for the synthesis of glycogen, and no genes for the formation of storage polysaccharides were identified. Nevertheless, the presence of several mannosyl transferases suggests that mannans, rather than glycogen, could serve as polysaccharide storage.

### 2.9. Sensitivity to Drugs

*Vickermania ingenoplastis* is sensitive to metronidazole and fexinidazole [14]. Both chemicals require enzyme-mediated reduction by hydrogenases and nitroreductases to generate cytotoxic species [38]. Genes encoding both enzymes, an iron-containing hydrogenase and a nitroreductase, were found in the analyzed genome.

### 2.10. Beta-Oxidation and Synthesis of Fatty Acids

The oxidation of fatty acids to carbon dioxide and water requires their activation through the linkage to coenzyme A. Subsequently, β-oxidation involves peroxisomes and mitochondria, whereby long-chain fatty acids are shortened first in the glycosomes, after which they are exported to the mitochondrion, where the resulting acetyl-CoA is further oxidized in the Krebs cycle either by acyl-CoA oxidase present in the glycosomes or by an isofunctional acyl-CoA dehydrogenase in the mitochondria [28]. The subsequent reactions are catalyzed by a single trifunctional enzyme in peroxisomes and by two separate enzymes in the mitochondria of most eukaryotes. These two latter enzymes are absent in *Vickermania* and *Phytomonas* spp. Thus, it is unlikely that a complete β-oxidation pathway is operational in *Vickermania*. Similarly, L-Leu cannot be oxidized to acetyl-CoA because isovaleryl-CoA dehydrogenase and 3-methylcrotonoyl-CoA carboxylase are absent.

However, the synthesis of fatty acids is possible due to the presence of genes encoding acetyl-CoA synthetase and acyl carrier protein. Since in all other trypanosomatids, type I fatty acid synthesis is absent, here it proceeds via elongase(s) that act on butyryl-CoA [39]. Several fatty acyl-CoA synthases (ligases) are present (Table S7). Of the four *L. major* fatty acyl dehydrogenases, only one is found in *V. ingenoplastis* (Table S8). Moreover, out of three other β-oxidation enzymes, namely enoyl CoA hydratase, 3-hydroxyacyl-CoA dehydrogenase and 3-ketoacyl-CoA thiolase, only the latter was identified. To oxidize the acetyl-CoA formed by β-oxidation, a functional Krebs cycle is required, as well. Therefore, complexes I and II are of vital importance for *Vickermania*. However, the absence of NAD-isocitrate dehydrogenase prevents the complete oxidation of acetyl-CoA to carbon dioxide and water, and, thus, *Vickermania* is likely unable to oxidize fatty acids to completion.

### 2.11. Amino Acid Metabolism

In other trypanosomatids, the amino acids Glu, Pro, and Thr may serve as energy sources in the absence of carbohydrates, such as glucose [28]. The presence of mitochondrial Glu and Pro dehydrogenases in the *V. ingenoplastis* genome suggests that it is also able to utilize these two amino acids. In contrast, most trypanosomatids rely on a catabolic Thr dehydrogenase to produce ammonia and 2-ketobutyrate, which is then irreversibly converted to propionyl-CoA and formate, Leishmaniinae and *Phytomonas* spp. use Thr dehydratase [28]. *Vickermania ingenoplastis* apparently uses the first pathway, which includes a mitochondrial Ser hydroxymethyltransferase, or Ser/Thr dehydratase.

The enzyme isovaleryl-CoA dehydrogenase participates in Val, Leu, and Ile degradation in other trypanosomatids [40]. It is absent in *V. ingenoplastis*, as well as kynureninase involved in the oxidation of Trp. Phe cannot be converted into Tyr because two of the three enzymes present in other trypanosomatids have been lost in both *Vickermania* and *Phytomonas* spp. [25] (Table S9). Similarly, a cobalamine-independent methionine synthase was absent from their genomes.

### 2.12. Catalase and Heme Synthesis

Catalase is present, yet it differs from its counterparts documented in Leishmaniinae [41,42] or *Blastocrithidia* spp. [43]. It remains to be investigated further why a gene encoding this important enzyme was acquired at least three times independently in the evolution of Trypanosomatidae.

All three heme-synthetic enzymes of prokaryotic origin (protoporphyrinogen oxidase, coproporphyrinogen III oxidase, and ferrochelatase) that are present in other Leishmaniinae [44] have been lost (or never acquired) in *V. ingenoplastis*.

## 3. Discussion

Trypanosomatids are famous for their remarkable adaptability to different environmental conditions. This is held as an explanation for the great variety of hosts (leeches and insects for the monoxenous, and arthropods, vertebrates, and plants for the dixenous parasites) that can be infected with these flagellates [5,6]. With the change of a host or switch between life cycle stages, trypanosomatids display unseen flexibility in metabolism [15,45]. Their insect-dwelling stages have an oxidative metabolism, in which amino and fatty acids serve as energy substrates. This is the case for the amastigotes of *Leishmania* or the epimastigotes and promastigotes of trypanosomes. All these stages require the presence of a fully active mitochondrion [46,47]. The other end of the spectrum is represented by aerobic fermentation of carbohydrates, with the mitochondrial metabolism reduced to a minimum. Examples of the latter are the bloodstream stages of African trypanosomes or their dyskinetoplastic cousins, *T. equiperdum* and *T. evansi* [48,49,50]. In the vast majority of trypanosomatids, such metabolic changes are reversible, and parasites go back and forth between an active and a less active mitochondrion during the switch of the life cycle stages. An exception to this rule is the plant trypanosomatids, belonging to the genus *Phytomonas* [51]. In these species, the switch back from aerobic fermentation to oxidative metabolism is blocked because several essential mitochondrial and nuclear metabolic proteins have been irreversibly lost. It was proposed that these deletions have occurred in order to make *Phytomonas* spp. insensitive to cyanide, which is present in plant tissues [25], an explanation implausible in the case of *V. ingenoplastis*, though.

Our genome analyses of *V. ingenoplastis* have confirmed the presence of most of the enzymes of carbohydrate metabolism, necessary for the production of all the major end-products under aerobic and anaerobic conditions [13] (Figure 1). Both *Phytomonas* spp. and *V. ingenoplastis* resemble each other in that they metabolize glucose to acetate, succinate and ethanol in varying amounts, depending on the oxygen availability. Ethanol production can be facilitated by isopropanol dehydrogenase (uniquely present in these species) converting acetaldehyde into ethanol [33,52]. However, *Phytomonas* and *Vickermania* differ in that *Phytomonas* excretes pyruvate and glycerol [53], similar to the bloodstream form of the African trypanosomes. In these species, glycerol is produced in the reversal of the glycerol kinase reaction via highly active glycosomal glycerol kinase [54]. *Vickermania ingenoplastis* lacks this enzyme (the annotated glycerol kinase is likely a xylulose kinase rather than a glycerol kinase). Instead, it excretes propionate using enzymes of the methylmalonyl-CoA pathway and the propionate cycle, which is absent in *Phytomonas* spp. (and in most other trypanosomatids).

In this work, we demonstrate that *V. ingenoplastis* is similar to *Phytomonas* spp. also in regard to the gene losses. This suggests that either these species belong to two closely related taxa, or they have been shaped by convergent evolution. *Vickermania ingenoplastis* resembles *Phytomonas* spp. not only because of the parallel loss of genes encoding subunits of complexes III and IV (questioning hypothesis that cyanide was a driving force of this process) but also because they lost numerous genes encoding enzymes involved in β-oxidation of fatty acids, the oxidation of aromatic, long or branched-chain amino acids. As an adaptation to these combined gene losses, *Phytomonas* spp. and *V. ingenoplastis* have drastically augmented their capacity for carbohydrate metabolism either by an increase in the copy number of glycolytic genes and/or the overall number of glycosomes in the cytosol [12,14,55]. Nevertheless, a critical difference between these two trypanosomatids is that *V. ingenoplastis* has increased its genome size by gene duplications, while *Phytomonas* spp. has done exactly the opposite [17,25].

Notably, *V. ingenoplastis* and *Phytomonas* spp. share a unique gene that encodes a Zn-containing NAD(+)-dependent alcohol dehydrogenase/isopropyl alcohol dehydrogenase (iPDH), an enzyme with a broad substrate specificity acting on primary and secondary alcohols [33,56]. In *Phytomonas*, the presence of iPDH facilitates the accumulation of ethanol as an end-product of its glycolysis [53]. Conversely, *V. ingenoplastis* excretes ethanol as one of the end-products of its carbohydrate metabolism [13]. The iPDH gene, not found in the majority of trypanosomatids, has been acquired via horizontal gene transfer from a bacterium and was proposed as a *Phytomonas*-specific marker enzyme [33,56]. The only other trypanosomatid possessing this gene (but as a pseudogene) is *Blechomonas ayalai* [28]. In conclusion, we postulate that *Vickermania* and *Phytomonas* are either closely related or convergently evolved trypanosomatids.

## 4. Materials and Methods

### 4.1. Genome Reassembly and Analysis of Its Completeness

The genome of *V. ingenoplastis* was reassembled using sequencing data reported previously [17] with MaSuRCA assembler v. 3.3.9 with the default settings, followed by two rounds of polishing using the assembler-associated Polca software [57]. This pipeline was not tested by us previously [17]. It resulted in a more contiguous assembly, which was used in the current study. For the assembly polishing, trimmed paired-end Illumina reads were mapped onto the genome assembly using the Burrows–Wheeler alignment tool (BWA) v. 0.7.17 with the default settings [58]. The assembly annotation was performed using a Companion server and the genome of *T. brucei* as a reference [59]. The basic assembly statistics and the completeness of the resulting assembly were assessed using QUAST v. 5.0.2 [60] and BUSCO v. 4.0.5 with the eukaryote_odb10 database [61], respectively.

### 4.2. Gene Expression Analysis

PolyA-enriched RNA library was sequenced on Illumina HiSeq 2500 platform with read length 151 bp, paired-end. Raw data available at NCBI SRA under BioProject accession number PRJNA675748 (SRR13015660). Sequencing reads were trimmed using Trimmomatic v. 0.39 [62] and mapped onto reference genome assembly with Bowtie2 v. 2.3.4.1 [63] with “—end-to-end—no-unal—sensitive” options. Read counts per gene were obtained with (for sorted bam file) and BEDtools [64]. Per gene RPKM, values were calculated using a custom python script.

### 4.3. Analysis of Metabolic Pathways

Metabolic pathways of *Vickermania ingenoplastis* were analyzed as described previously [28,65], using “all against all” BLASTp searches with an E-value cutoff of 10^−20^ and previously published (but reassembled) genomic data [17]. In some cases, a stricter E-value cutoff of 10^−50^ was used in order to distinguish between true orthologous proteins and more distant homologs, which are not necessarily functional orthologues.

## Data Availability

The data presented in this study are openly available in NCBI SRA under BioProject accession number PRJNA675748 (SRR13015660).

## Supplementary Materials

The following are available online at https://www.mdpi.com/2076-0817/10/1/68/s1, Table S1: *Leishmania major* proteins not found in *Vickermania ingenoplastis* genome by BLASTp (hereafter, threshold 10^−20^). Table S2: Presence/absence for orthologues of 458 genes encoding *L. major* metabolic proteins in *V. ingenoplastis* by BLASTp. Expression (RNA-seq data) is indicated as RPKM values, and Expression level (over genome). The color coding for the expression level is green (below 5%), yellow (5–25%), orange (25–75%), and red (over 75%). Table S3: Copy numbers of genes coding for metabolic enzymes in *V. ingenoplastis*. Table S4: Enzymes unique to *V. ingenoplastis* (absent from *L. major* genome) after exclusion of hypothetical or viral proteins. Table S5: Subunits of the respiratory chain complexes in *L. major*, *T. brucei*, and *V. ingenoplastis*. Table S6: Enzymes of the carbohydrate metabolism in *L. major* and *V. ingenoplastis*. Genes absent in *V. ingenoplastis* are highlighted in red. Table S7: Enzymes of fatty acid oxidation in *B. saltans*, *L. major*, *T. brucei*, and *V. ingenoplastis*. Table S8: Enzymes present in *L. major* but absent in both *V. ingenoplastis* and *Phytomonas* spp. Table S9: Enzymes of other pathways.
